# Optical coherence tomography in neurodegenerative disorders

**DOI:** 10.1590/0004-282X-ANP-2021-0134

**Published:** 2022-03-15

**Authors:** Leonardo Provetti CUNHA, Leopoldo Antônio PIRES, Marcelo Maroco CRUZEIRO, Ana Laura Maciel ALMEIDA, Luiza Cunha MARTINS, Pedro Nascimento MARTINS, Nadia SHIGAEFF, Thiago Cardoso VALE

**Affiliations:** 1 Universidade Federal de Juiz de Fora, Faculdade de Medicina, Divisão de Oftalmologia, Juiz de Fora MG, Brazil. Universidade Federal de Juiz de Fora Faculdade de Medicina Divisão de Oftalmologia Juiz de Fora MG Brazil; 2 Universidade de São Paulo, Faculdade de Medicina, Divisão de Oftalmologia, São Paulo SP, Brazil. Universidade de São Paulo Faculdade de Medicina Divisão de Oftalmologia São Paulo SP Brazil; 3 Universidade Federal de Juiz de Fora, Faculdade de Medicina, Pós-Graduação em Saúde, Núcleo de Pesquisa em Neurologia, Juiz de Fora MG, Brazil. Universidade Federal de Juiz de Fora Faculdade de Medicina Pós-Graduação em Saúde Juiz de Fora MG Brazil; 4 Universidade Federal de Juiz de Fora, Hospital Universitário, Serviço de Neurologia, Juiz de Fora MG, Brazil. Universidade Federal de Juiz de Fora Hospital Universitário Serviço de Neurologia Juiz de Fora MG Brazil; 5 Universidade Federal de Juiz de Fora, Faculdade de Medicina, Departamento de Clínica Médica, Juiz de Fora MG, Brazil. Universidade Federal de Juiz de Fora Faculdade de Medicina Departamento de Clínica Médica Juiz de Fora MG Brazil; 6 Universidade Federal de Juiz de Fora, Instituto de Ciências Humanas, Departamento de Psicologia, Juiz de Fora MG, Brazil. Universidade Federal de Juiz de Fora Instituto de Ciências Humanas Departamento de Psicologia Juiz de Fora MG Brazil

**Keywords:** Tomography, Optical Coherence, Alzheimer Disease, Parkinson Disease, Multiple Sclerosis, Neurodegenerative Diseases, Amyotrophic Lateral Sclerosis, Retina, Tomografia de Coerência Óptica, Doença de Alzheimer, Doença de Parkinson, Esclerose Múltipla, Doenças Neurodegenerativas, Esclerose Amiotrófica Lateral, Retina

## Abstract

Structural imaging of the brain is the most widely used diagnostic tool for investigating neurodegenerative diseases. More advanced structural imaging techniques have been applied to early or prodromic phases, but they are expensive and not widely available. Therefore, it is highly desirable to search for noninvasive, easily accessible, low-cost clinical biomarkers suitable for large-scale population screening, in order to focus on making diagnoses at the earliest stages of the disease. In this scenario, imaging studies focusing on the structures of the retina have increasingly been used for evaluating neurodegenerative diseases. The retina shares embryological, histological, biochemical, microvascular and neurotransmitter similarities with the cerebral cortex, thus making it a uniquely promising biomarker for neurodegenerative diseases. Optical coherence tomography is a modern noninvasive imaging technique that provides high-resolution two-dimensional cross-sectional images and quantitative reproducible three-dimensional volumetric measurements of the optic nerve head and retina. This technology is widely used in ophthalmology practice for diagnosing and following up several eye diseases, such as glaucoma, diabetic retinopathy and age-related macular degeneration. Its clinical impact on neurodegenerative diseases has raised enormous interest over recent years, as several clinical studies have demonstrated that these diseases give rise to reduced thickness of the inner retinal nerve fiber layer, mainly composed of retinal ganglion cells and their axons. In this review, we aimed to address the clinical utility of optical coherence tomography for diagnosing and evaluating different neurodegenerative diseases, to show the potential of this noninvasive and easily accessible method.

## RESUMO

A avaliação estrutural do cérebro, feita por meio dos exames de neuroimagem, é a forma mais utilizada de ferramenta diagnóstica e de acompanhamento das doenças neurodegenerativas. Técnicas de imagem mais sofisticadas podem ser necessárias especialmente nas fases mais precoces, antes mesmo do surgimento de quaisquer sintomas, porém costumam ser caras e pouco acessíveis. Sendo assim, é de fundamental importância a busca de biomarcadores não invasivos, de fácil acesso e baixo custo, que possam ser utilizados para rastreio populacional e diagnóstico mais precoce. Nesse cenário, o número de estudos com ênfase em técnicas de imagem para avaliação estrutural da retina em pacientes com doenças neurodegenerativas tem aumentado nos últimos anos. A retina apresenta similaridade embriológica, histológica, bioquímica, microvascular e neurotransmissora com o córtex cerebral, tornando-se assim um biomarcador único e promissor nas doenças neurodegenerativas. A tomografia de coerência óptica é uma moderna técnica de imagem não invasiva que gera imagens seccionais bidimensionais de alta resolução e medidas volumétricas tridimensionais reprodutivas do disco óptico e da mácula. Essa tecnologia é amplamente utilizada na prática oftalmológica para o diagnóstico e o seguimento de diversas doenças oculares, como glaucoma, retinopatia diabética e degeneração macular relacionada à idade. A redução da espessura da camada de fibras nervosas da retina e das camadas de células ganglionares em pacientes com doenças neurodegenerativas foi demonstrada em diversos estudos clínicos nos últimos anos. Nesta revisão, abordamos as principais aplicações clínicas da tomografia de coerência óptica nas doenças neurodegenerativas e discutimos o seu papel como potencial biomarcador nessas afecções.

## INTRODUCTION

Neurodegenerative diseases are conditions that can affect the central and peripheral nervous systems, leading to cognitive, motor, speech and even respiratory impairment^1^. These age-related disorders have become increasingly prevalent with the aging of the population worldwide over recent years^1^. Mild cognitive impairment (MCI), Alzheimer’s disease (AD), multiple sclerosis (MS), Parkinson’s disease (PD) and amyotrophic lateral sclerosis (ALS) have distinct pathophysiological mechanisms, but share progressive neuronal damage, leading to focal or overall loss of functions^2^.

The most widely used diagnostic tool for investigating structural changes in the brains of patients with neurodegenerative diseases is magnetic resonance imaging (MRI)^3^. Despite its ability to detect morphological and volumetric changes in these patients, the diagnostic sensitivity of MRI is higher when the clinical disease is well-established. More advanced structural imaging techniques such as positron emission tomography, diffusion-weighted imaging and diffusion tensor imaging, magnetic spectroscopy and perfusion imaging have been applied in the early or prodromic phases of neurodegenerative diseases^3^. However, these imaging techniques are expensive, invasive and difficult to access, especially in developing countries, which makes them unsuitable for populational screening. Therefore, it is highly desirable to search for noninvasive, easily accessible, low-cost clinical biomarkers suitable for large-scale population screening, in order to focus on making diagnoses at the earliest stages of the disease. In this scenario, imaging studies focusing on the structures of the retina have increasingly been used for evaluation of neurodegenerative diseases. The retina shares embryological, histological, biochemical, microvascular and neurotransmitter similarities with the cerebral cortex, thus making it a uniquely promising biomarker for neurodegenerative diseases^4^.

**Figure 1.**
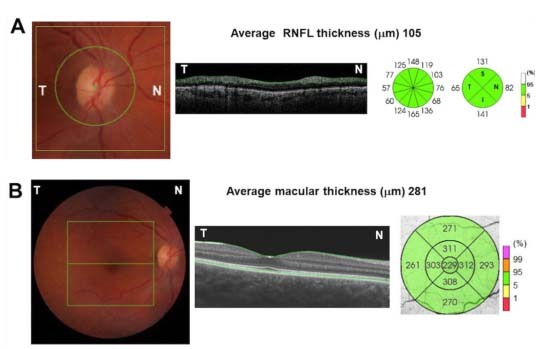
Schematic representation of the optic nerve head (ONH) and macula using spectral-domain optical coherence tomography (OCT) scanning protocols on the right eye of a normal individual. (A) 3D optic disc report, showing the ONH. Left panel: 6 × 6 mm scanned area centered on the ONH (green square) and the 3.4 mm peripapillary analyzed area for assessment of RNFL thickness. Central panel: OCT B-scan representing the cross-sectional retinal image around the ONH. The boundaries of the RNFL are represented by green lines. Right panel: Schematic representation of the peripapillary RNFL thickness measurements divided into 4 and 12-clock hour sectors with the values in microns. (B) 3D macula report, showing the macular area. Left panel: 6 × 6 mm scanned area centered on the fovea (green square). The horizontal green line represents the scanned horizontal central area. Central panel: Horizontal OCT B-scan representing the cross-sectional retinal image. The boundaries of the internal limiting membrane and the retinal pigment epithelium are represented by green lines. Right panel: ETDRS map divided in 9 sectors with the respective values of the total macular thickness measurements in microns.

**Figure 2.**
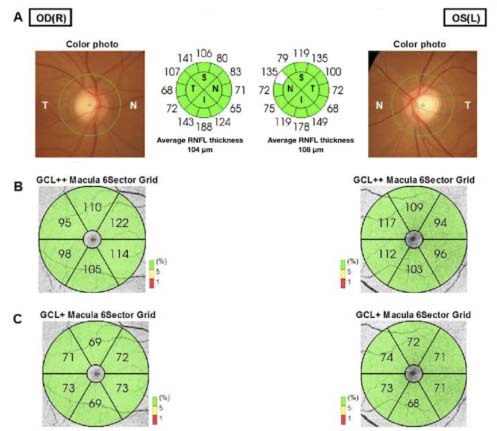
Swept-source optical coherence tomography (OCT) 3D wide disc and macula report (12 × 9 mm), on OCT three-dimensional images of both eyes (OU) of a 45-year-old healthy woman. (A) Peripapillary retinal nerve fiber (pRNFL) layer thickness measurements of OU. Average pRNFL thickness measurements are within normal limits: 104 microns in right eye (OD) and 108 microns in left eye (OS). (B) Ganglion cell complex (GCL++) macula thickness map divided into six sectors. GCL++ thickness measurements are within normal limits (in green) in OU. (C) Ganglion cell/inner plexiform layer (GCL+) macula thickness map divided into 6 sectors. GCL+ thickness measurements are within normal limits (in green) in OU. In this example, OCT thickness measurements did not demonstrate any signs of axonal loss and neuronal loss.

Optical coherence tomography (OCT) is a modern noninvasive imaging technique that provides high-resolution two-dimensional cross-sectional images and quantitative reproducible three-dimensional volumetric measurements of the optic nerve head and retina^5^ (Figures 1 and 2). This technology is widely used in ophthalmology practice for diagnosing and following up several eye diseases, such as glaucoma, diabetic retinopathy and age-related macular degeneration. Its clinical impact on neurodegenerative diseases has raised enormous interest over recent years, as several clinical studies have demonstrated that these diseases give rise to reduced thickness of the inner retinal nerve fiber layer (RNFL), mainly composed of retinal ganglion cells and their axons^6^.

T: temporal, N: nasal, RNFL: retinal nerve fiber layer, mm: microns; ETDRS: early treatment diabetic retinopathy study.

Due to the similarities of the microvascular structures of the retina and the brain, and also because of the presence of vascular abnormalities of the brain in many patients with neurodegenerative disorders, changes to retinal vascular density might also have potential as an ocular biomarker for neurodegenerative conditions. OCT-angiography (OCTA) is a noninvasive technique for imaging the microvasculature of the retina, in which light laser reflectance is used to detect the movement of intravascular red blood cells and thus reconstruct the retinal microvasculature in detail, without the use of contrast^7^.

In this review, we aimed to address the clinical utility of OCT and OCTA for diagnosing and evaluating different neurodegenerative diseases, in order to show the potential of this noninvasive and easily accessible method.

## PRINCIPLES OF OPTICAL COHERENCE TOMOGRAPHY

OCT is a noninvasive diagnostic technique that generates in vivo cross-sectional images of the retina. It uses near-infrared light, based on low-coherence interferometry, to create a cross-sectional image of the retina. The first commercially available versions of OCT used time-domain (TD-OCT) technology, which requires long acquisition times and provides axial and lateral resolutions of 15 mm. Improvements have been achieved over the past two decades with the emergence of spectral-domain OCT (SD-OCT) technology. This provides three-dimensional high-resolution cross-sectional retinal images with an axial resolution up to five times greater and imaging speeds approximately 60 times greater than TD-OCT^8^.

The retinal layers are automatically identified by the OCT device. It considers the differences in reflectivity and signals generated by each retinal layer. Thus, peripapillary RNFL (pRNFL) is defined as the distance between the internal limiting membrane and the retinal ganglion cell/inner plexiform layer. These layers are located between the RNFL and the inner nuclear layer. The total macular thickness is calculated considering the distance between the internal limiting membrane and the retinal pigment epithelium^8^.

The OCT device automatically estimates the pRNFL and the macula thickness measurements. In most OCT devices, pRNFL thickness measurement reports are obtained through a 6 × 6 mm scanned area centered on the optic nerve head, consisting of 521 A-scans horizontally and 256 vertically. The measured area consists of a 3.4 mm diameter circle centered on the optic nerve head (Figure 1A). The measurements made are the average thickness, the thicknesses of the four quadrants (superior, temporal, inferior and nasal) and the thicknesses of the 12 clock hour segments (in mm). The macular analysis protocols consist of a scanned area of 6 × 6 mm, with 512 A-scans horizontally and 128 vertically. The macular analysis is based on a 6 mm circular map divided into a nine-segment map. The measurements made are the average macular thickness and the thickness of each of the nine sectors (in mm)^8^ (Figure 1B).

## OPTICAL COHERENCE TOMOGRAPHY IN MILD COGNITIVE IMPAIRMENT

MCI is recognized as a possible intermediate phase between senescence and dementia that denotes the presence of subjective and mild complaints of cognitive impairment, compared with healthy older people, without impacting the performance of activities of daily living^9^. Individuals in whom memory is one of the impaired domains (amnestic MCI -aMCI) have higher conversion rates to dementia. This is also seen when there is an association with vascular and parkinsonian symptoms^10^. Given the difficulties in clinically differentiating aMCI from the early stages of AD, an increasing number of studies have aimed to enable a more precise and earlier etiological diagnosis using techniques such as neuroimaging and serum/cerebrospinal fluid biomarkers^10^. In this context, OCT may play a role as another potential biomarker.

Several previous studies have indicated that MCI patients present decreased pRNFL thickness^11^^,^^12^^,^^13^. Compared with AD patients, the reduction in pRNFL thickness seems to be less pronounced in MCI patients, thus suggesting that a direct correlation exists between the severity of the disease and the amount of axonal impairment. These findings are in accordance with a recently published study by our group^14^. In this study, we found that most OCT parameters were significantly lower in individuals with aMCI, especially the macular ganglion cell complex thickness measurements. Moreover, the macular thickness parameters were significantly correlated with the severity of cognitive impairment^14^.

Some recent previous studies showed changes in the retinal microvascular network in individuals with aMCI, with significant reductions in vessel density in the superficial capillary plexus and deep capillary plexus, in addition to decreased blood flow^15^^,^^16^^,^^17^. These studies also showed that parafoveal and peripapillary densities were positively correlated with low scores in the Montreal Cognitive Assessment (MoCA). The reductions in both vessel and perfusion densities of the superficial capillary plexus seen in OCTA have been positively correlated with measurements of brain volume using volumetric MRI, in individuals with MCI and AD^17^.

## OPTICAL COHERENCE TOMOGRAPHY IN ALZHEIMER’S DISEASE

AD is the most common neurodegenerative disorder in the elderly. Visual abnormalities occur frequently among AD patients and include decreased perception of contrast and movement, reduction of color vision and even loss of vision^18^. These abnormalities may be due to disorders in primary areas, notably the primary visual cortex^19^^,^^20^. Nevertheless, several studies have shown signs of specific impairment of the retina and the optic nerve in AD patients^21^^,^^22^^,^^23^.

A wide range of studies have shown RNFL thinning in AD, in comparison with controls^11^^,^^12^^,^^22^^,^^24^^,^^25^^,^^26^^,^^27^^,^^28^^,^^29^^,^^30^^,^^31^. In general, the pRNFL thickness is diffusively decreased, affecting all sectors around the optic disc, which suggests that the axonal loss in AD patients results from a diffuse degenerative process in the ganglion cell layer^11^^,^^24^^,^^27^^,^^28^^,^^29^.

**Figure 3.**
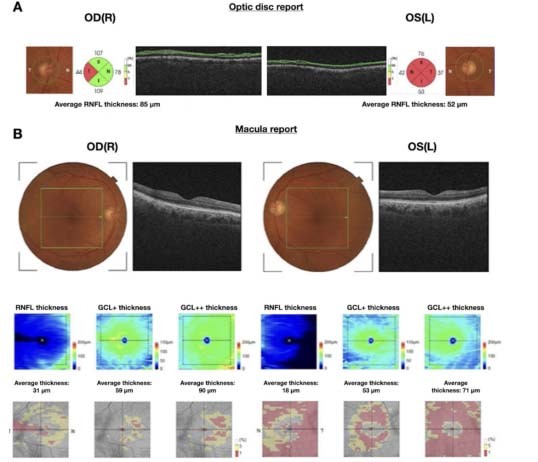
Example of optical coherence tomography (OCT) 3D optic disc and macula report, on OCT three-dimensional images of both eyes (OU) of a 75-year-old male with a three-year diagnosis of Alzheimer’s disease and Mini-Mental State Examination test score of 15/30. (A) Optic disc report (6 × 6 mm) of OU, including total average peripapillary retinal nerve fiber layer (RNFL) measurements (in microns). Note the values outside of normal limits in the temporal quadrant in the right eye and in all four quadrants in the left eye (in red). (B) Inner macular thickness report (7 × 7 mm) of macular RNFL, ganglion cell/plexiform layer (GCL+) and ganglion cell/plexiform layer plus RNFL (GCL++) thickness measurements (in microns). Note the values outside of normal limits (in red) for all three parameters in OU, thus showing greater severity of neuronal loss in the left eye.

In 2006, Iseri et al.^25^ were the first to evaluate the total macular thickness, in 28 eyes from 14 AD patients. They showed that these patients had significantly reduced macular thickness in the nasal, temporal and inferior fields, as well as reduced total macular volume. Moschos et al.^31^ showed that the inner macular sectors are the ones most affected. This was supported by data from a study by Cunha et al.^24^, in which 45 eyes from 24 AD patients were included. That study showed thickness reductions in all sectors, except for the outer inferior sector, occurring preferentially in the inner macular areas. The ganglion cell layer and its axons contribute approximately one third of the total retinal thickness in the macula and seem to be the layer most affected in AD, according to previous tomographic and histopathological studies^21^^,^^23^^,^^24^^,^^32^. Recent technological enhancements of OCT have allowed greater segmentation of the macular retinal layers, consequently allowing the inner layers to be studied with greater precision. Assessment of the inner retinal layer is crucial, mainly because these layers (i.e. the macular RNFL and ganglion cell/inner plexiform layers) reflect the neuronal loss in patients with AD (Figure 3).

Some studies have found a correlation between OCT findings in AD patients and results from the Mini-Mental State Examination (MMSE)^25^^,^^30^. Cunha et al.^24^^,^^32^ showed that greater pRNFL thinning, denoting axonal loss, and lower total macular and ganglion cell layer thicknesses, representing neuronal loss, were correlated with worse cognitive performance in the MMSE. Hence, OCT can be useful not only for assessing neuronal and axonal loss in AD patients, but also for measuring the cognitive decline in these patients.

A recent meta-analysis, involving 30 cross-sectional studies with 1257 AD patients, 305 MCI patients and 1460 controls^6^, confirmed the association between OCT-based retinal measurements of the ganglion cell-inner plexiform layer, ganglion cell complex, macular volume and thickness and choroidal and RNFL thicknesses of AD patients, which were all significantly different from the controls.

OCTA can also be helpful in assessing retinal changes in AD, as shown in an increasing number of studies. Using OCTA, Bulut et al.^33^ were the first to show decreased vascular density in the whole macular, foveal and parafoveal zones in AD patients, compared with controls, along with enlargement of the foveal avascular zone. Other authors have observed that OCTA findings correlate with cognitive function and that overall vascular density is lower in AD patients than in controls^34^^,^^35^.

In addition, one of the first uses of OCT as a potential biomarker was among adults with preclinical AD. In 2018, using SD-OCT macular parameters, Santos et al.^36^ conducted a 27-month longitudinal study on midlife adults with preclinical AD with multiple risk factors. In comparing them with healthy control subjects, significant reductions in macular RNFL and in the outer nuclear layer and inner plexiform layer volumes were noted over the follow-up period. This was one of the first reports on neuronal layer volumetric changes assessed by means of OCT in subjects with preclinical neurodegenerative disease, and it highlighted the potential use of OCT as biomarker.

## OPTICAL COHERENCE TOMOGRAPHY IN MULTIPLE SCLEROSIS

MS is the most prevalent chronic inflammatory disease of the central nervous system (CNS)^37^. Visual loss is one of the most frequent symptoms and is typically related to retrobulbar optic neuritis (ON), which is characterized by sudden onset of visual loss accompanied by eye pain that is worsened or triggered by eye movements^38^.

In the acute phase, OCT in ON can be useful for detecting and quantifying optic disc edema, which is observed through increased pRNFL thickness. In accordance with a previous study, the mean average pRNFL thickness in patients with ON secondary to MS is 113 µm, ranging from 87 to 271 µm^39^. If these measurements are greater than 270 µm, it indicates very pronounced edema, which would be atypical for MS. OCT can also be used to monitor resolution of edema and to evaluate macular edema or serous retinal detachment, which are typical of other inflammatory conditions such as neuroretinitis and posterior or postoperative uveitis.

Another important application of OCT is in detection of subsequent axonal loss after the outbreak of ON^40^. A systematic review and meta-analysis on OCT-based retinal layer atrophy measurements in MS showed that RNFL is thinner in the peripapillary and macular regions in MS patients with ON and without ON, compared with controls^41^. There was no statistical difference in the thickness of the combined outer nuclear layer and outer plexiform layer when MS patients were compared with controls. Quantitative layer segmentation data showed that inner retinal layer atrophy was severe after ON and was also significantly reduced in the eyes of patients with MS who had never had ON, compared with control eyes. It has been shown that a single episode of ON does not imply greater risk of a progressive decrease in RNFL thickness, thus suggesting that thinning might occur as a result of recurrent episodes of ON^42^. However, axonal loss and reduction of pRNFL thickness can occur regardless of a previous history of ON, thus denoting the existence of subclinical axonal loss, possibly linked to irreversible damage caused by the neurodegenerative nature of MS^40^^,^^41^.

The reduction in the thickness of the pRNFL is most evident around three months after an ON episode and tends to stabilize around 12 months. However, longitudinal studies have shown an annual atrophy rate of -1.4 µm/year: higher in MS patients with ON (-0.91 µm/year) than in MS patients without ON (-0.53 µm/year)^41^.

Another important matter is the association of axonal loss (pRNFL thickness) and neuronal loss (macular ganglion cell complex thickness) with the intensity of visual functional loss, measured in terms of visual acuity, computerized visual field examination and color vision. Patients with greater axonal and neuronal loss after an ON outbreak are the ones who are expected to present the greatest impairment of visual function^43^.

Endothelial dysfunction, probably secondary to inflammation, and a chronic state of impaired CNS venous drainage seem to play an important role in the development and course of MS^44^. There is emerging evidence that OCTA may serve as an effective tool for detecting pathological alterations that occur in the retinal vasculature of patients with MS. Several studies using OCTA have shown that the retinal vessel density of both macular and peripapillary areas were reduced in MS patients^45^^,^^46^^,^^47^^,^^48^. Thus, OCTA has the ability to detect subclinical vascular changes and is a potential biomarker for diagnosing MS and measuring its progression.

## OPTICAL COHERENCE TOMOGRAPHY IN PARKINSON’S DISEASE

PD is the most common type of parkinsonism and the second most common neurodegenerative disease affecting the elderly population. The ocular changes that have been described in PD include visual dysfunction (visual acuity, dynamic contrast sensitivity and color discrimination), pupil abnormality, lens opacity, motion perception, visual processing speeds, facial recognition problems, chronic visual hallucinations and retinal neuronal loss and dysfunction^49^^,^^50^. These visual disorders are thought to be related to α-synuclein deposition and dopamine deficiency in the retina, thus mirroring the defining pathological features of PD in the brain^49^^,^^50^. Alpha-synuclein is an abundant neuronal protein that regulates synaptic vesicle trafficking and subsequent neurotransmitter release. When aggregated, it forms insoluble fibrils known as synucleinopathies, under pathological conditions, and this may lead to various cellular disorders. Dopamine is released by a unique set of amacrine cells of the inner nuclear layer and activates D1 and D2 dopamine receptors, which are distributed throughout the retina. Reduction in retinal dopamine has mostly been correlated with reduced color vision, visual contrast sensitivity and visual acuity^51^. Overall, visual impairment has been considered to be a consequence of PD progression, but a number of visual features can be observed in early PD and could even be present in the prodromal phase.

Recent advances have led to increasing interest in the role of the retina as a potential biomarker for making an early diagnosis of PD, and also as a means of measuring disease progression and evaluating novel therapeutic strategies. If early dopamine dysfunction leads to retinal structural abnormalities that could be detected through imaging of the retina, OCT could serve as a potential biomarker for making early diagnoses and prognoses in PD.

In 2004, Inzelberg et al.^52^ were the first to show RNFL thinning through OCT, in ten PD patients. Subsequent studies confirmed their findings and also showed reductions in macular volume and thickness^53^^,^^54^. However, several other studies reported that RNFL thickness was similar in patients and controls^55^^,^^56^^,^^57^. The akinetic-rigid subtype of PD was found to have thinner RNFL than the tremor-predominant subtype^58^, and the thickness of the RNFL was found to have a negative correlation with the severity of PD measured according to the Hoehn-Yahr stage and the Unified Parkinson’s Disease Rating Scale (UPDRS) scores^55^^,^^56^^,^^59^. RNFL thickness has also been correlated with cognition^60^ and the presence of hallucinations^57^. Recent longitudinal studies have shown a progressive decrease in RNFL thickness, and this was accompanied by progressive visual dysfunction^61^^,^^62^.

A segmented retinal layer measurement might provide better knowledge of retinal thinning in PD. A recent meta-analysis demonstrated that the ganglion cell layer and inner plexiform layer are the macular layers most affected in PD^63^. Changes to the retina in PD patients suggest vascular and dopaminergic mechanisms, but further studies are needed in order to support this theory. Although there are many challenges regarding OCT assessments in PD patients, pooled data from a recent systematic review confirmed that robust associations between retinal OCT measurements and PD exist^64^, thus emphasizing the usefulness of OCT as a potential imaging biomarker in PD. Moreover, combination of OCT with OCTA may yield better diagnostic ability than either of these alone, hence providing additional biomarker methods for measuring PD onset and progression^65^.

## OPTICAL COHERENCE TOMOGRAPHY IN AMYOTROPHIC LATERAL SCLEROSIS

ALS is a neurodegenerative disease that classically affects the corticospinal tract and the motor neurons in the anterior horn of the spinal cord, and it is the most common motor neuron disease. Although it is essentially a motor disease, non-motor symptoms are common and may even precede the motor symptoms. About 10 to 75% of the patients with this disease have cognitive impairments and 15 to 41% of the cases progress to dementia^66^.

Non-motor nervous system involvement in ALS has been demonstrated through neuroimaging, electrophysiological and histopathological tests. These draw attention to possible involvement of the anterior optic pathway and, more specifically, of the retina and the optic nerve^67^^,^^68^^,^^69^^,^^70^. Histopathological analysis on ALS eyes and mice with the UBQLN2(P497H) mutation, as well as OCT measurements, showed findings of intraretinal deposits and axonal loss, which were supportive of involvement of the anterior visual pathway^71^.

In a study on 20 ALS patients and 25 matched healthy controls, the average pRNFL thickness found to be significantly reduced in ALS patients, compared with controls (102.57±13.46 compared with 97.11±10.76; p=0.04). A positive correlation was also found between the functional abilities of ALS patients, as assessed using the ALS Functional Rating Revised Scale (ALSFRS-R), and the average pRNFL thickness in most quadrants^72^. In a follow-up study on ALS patients, Rojas et al.^73^ showed that in ALS follow-up patients, compared with ALS baseline patients, there was significant macular thinning in the inner and outer macular ring in inferior areas and significant RNFL thinning in the superior and inferior quadrants in the follow-up patients. Another interesting correlation was found between RNFL thickness and fractional anisotropy measurements of the corticospinal tract in a diffusion tensor imaging study^74^, which showed that in fact retinal involvement is associated with overall neurodegeneration in ALS. Fractional anisotropy is a useful measurement of connectivity in the brain, derived from the diffusion tensor imaging data. It measures the degree of anisotropy of water molecules and allows inferences regarding alterations to the axonal diameter, fiber density or myelin structure.

In addition to RNFL abnormalities, other changes to retinal layer thicknesses and retinal blood vessels have also been described in ALS^75^. The outer and inner nuclear layers were found to be significantly thinner in ALS patients than in controls, and the outer wall thickness of retinal vessels was significantly thicker in ALS patients. Interestingly, no study reported any thinning of the ganglion cell layer, which has been extensively studied due to its relationship with optic nerve damage in other diseases such as AD and PD^75^. OCT could probably not only serve as a biomarker and progression marker tool, but also provide a new opportunity to delve into the pathogenesis of the disease.

## OPTICAL COHERENCE TOMOGRAPHY IN OTHER NEURODEGENERATIVE DISEASES

OCT has been studied in Friedreich’s ataxia (FRDA), the most common autosomal recessive ataxia worldwide, and in the rarer ataxia syndrome of autosomal recessive spastic ataxia of Charlevoix-Saguenay (ARSACS). Patients with FRDA may have a measurable degree of pRNFL thinning, as determined using OCT, which can be a useful tool for following up these patients^76^. ARSACS is a rare neurodegenerative disorder caused by mutations in the SACS gene, and thickened retinal nerve fibers seen on fundoscopy form part of the clinical picture. In this regard, OCT appears to be a more sensitive and specific means for detecting RNFL thickness. In a large cohort of genetically confirmed ataxia cases, all 17 ARSACS patients were visually asymptomatic and did not have any previous history of ophthalmological complaints, and all of them presented pRNFL thickness loss on OCT, whereas this finding was detected via fundoscopy only in 12 of them. This is a useful tool for identifying cases of ARSACS among other causes of ataxia^77^.

Adrenoleukodystrophy (ALD) is a disease linked to the X chromosome that presents with different neurological phenotypes, ranging from very severe cerebral forms to less severe adrenomyeloneuropathy. Progressive myelopathy is the main cause of disability in these patients. However, the visual system may be involved and neurodegeneration of the spinal cord in ALD has been correlated with pRNFL thickness^78^. In a cross-sectional and longitudinal study on 11 symptomatic adult ALD males, Bianchi-Marzoli et al.^79^ showed that OCT can reveal retinal abnormalities in the most disabled patients, particularly in the inferior pRNFL and inner macula.

OCT has also been used in relation to other neurodegenerative movement disorders, such as Wilson’s disease, Huntington’s disease and atypical parkinsonian syndromes. In Wilson’s disease, which is an inherited autosomal recessive disorder that leads to pathological copper accumulation in different organs, thinning of the pRNFL and macular thickness has been detected via OCT^80^, especially in patients with brain imaging abnormalities^81^. A significant negative correlation was found between OCT parameters and neurological impairment according to a specific scale for patients with Wilson’s disease^82^. Eye movement disorders are key clinical features in Huntington’s disease. OCT performed on 26 Huntington’s disease patients showed that RNFL thickness was significantly reduced, compared with controls, and there was a significantly negative correlation with disease duration. Macular volume also correlated negatively with disease duration and motor scores^83^. Another cross-sectional study on eight Huntington’s disease patients showed that both choroidal and retinal macula were altered in the disease^84^. Other studies followed^85^^,^^86^, including an OCTA study^87^, which affirmed the usefulness of OCT as a potential biomarker of neurodegeneration in Huntington’s disease patients. Studies on multiple system atrophy and progressive supranuclear palsy have shown conflicting results, but there is evidence of RNFL thickness reduction, also correlated with disease severity, and to a worse extent than in PD patients^88^.

Cerebral autosomal dominant arteriopathy with subcortical infarcts and leukoencephalopathy (CADASIL) is considered to be a genetic form of small-vessel disease that causes subcortical dementia. In a cross-sectional study involving 17 CADASIL patients, RNFL thickness was significantly reduced, compared with controls^89^. In a more common form of dementia, frontotemporal dementia, OCT can also be useful, considering that RNFL thickness reduction has been shown^90^.

Lastly, OCT has been evaluated as a potential optical guidance system for deep brain stimulation. The results showed that catheter-based OCT had the resolution and contrast necessary for targeting. Evidence has also been provided to show that vacuoles in spongiform encephalopathies are another structure that OCT can detect with high contrast^91^. This exemplifies the possible future clinical applications of OCT in disorders that goes beyond the visual system.

## LIMITATIONS AND CONCERNS

Over the last decade, there has been significant focus on research addressing OCT applications for diagnosing and following up many neurodegenerative diseases. Table 1 summarizes the main findings from the most important papers on OCT use in MCI, AD, MS, PD and ALS. Use of OCT to define a biomarker may have an impact on population screening for neurodegenerative diseases, especially among patients with risk factors, given that it is an easily accessible and minimally invasive test. Another important clinical application of OCT is in evaluating the therapeutic effects of certain drugs on ocular parameters and the possibility of prevention of neuronal degeneration in these patients. To cite an example, Mello et al.^92^ compared the macular and choroidal thickness parameters in patients with PD with or without treatment using pramipexole, a dopamine agonist. Their results showed that thinning of many macular parameters occurred, especially macular RNFL and the ganglion cell layer + inner plexiform layer, in patients with PD without pramipexole treatment, compared with those who were using pramipexole. Their findings suggested that pramipexole treatment seems to prevent retinal degeneration in PD.

**Table 1.** Main optical coherence tomography and optical coherence tomography-angiography findings in neurodegenerative disorders.

Structural parameters	Main findings	Key references
Mild cognitive impairment		
pRNFL	Progressively thinned from controls to mild cognitive impairment and from mild cognitive impairment to Alzheimer’s disease	Lee et al.^11^, Paquet et al.^12^, Gao et al.^13^, Almeida et al.^14^
Macula	Decreased ganglion cell complex thickness, compared with controls	Almeida et al.^14^
	Correlation between OCT abnormalities and cognitive impairment	Almeida et al.^14^
Microvascular	Decreased vessel density and blood flow correlated with lower MoCA scores	Zhang et al.^15^, Criscuolo et al.^16^, Yoon et al.^17^
Alzheimer’s disease		
pRNFL	Diffuse thickness reduction, compared with healthy controls	Chan et al.^6^, Lee et al.^11^, Paquet et al.^12^, Parisi et al.^22^, Cunha et al.^24^, Iseri et al.^25^, Kesler et al.^26^, Kirbas et al.^27^, Lu et al.^28^, Marziani et al.^29^, Moreno-Ramos et al.^30^, Moschos et al.^31^
	Correlation between thinning and lower MMSE results	Cunha et al.^24^, Iseri et al.^25^, Moreno-Ramos et al.^30^, Cunha et al.^32^
Macula	Diffuse thinning, especially in the ganglion cell complex	Chan et al.^6^, Lee et al.^11^, Cunha et al.^24^, Kirbas et al.^27^, Lu et al.^28^, Marziani et al.^29^, Moschos et al.^31^
	Reduced volume, compared with controls	Chan et al.^6^, Iseri et al.^25^
	Correlation with MMSE results	Cunha et al.^24^, Iseri et al.^25^, Moreno-Ramos et al.^30^, Cunha et al.^32^
	Decrease in macular RNFL, outer nuclear layer and inner plexiform layer volumes, in preclinical AD relative to controls	Santos et al.^36^
Microvascular	Decreased vascular density	Bulut et al.^33^, Zhang ^34^, Song ^35^
	Foveal avascular zone enlargement	Bulut et al.^33^
	Correlation with cognitive function	Bulut et al.^33^, Zhang et al.^34^, Song et al.^35^
Multiple sclerosis		
pRNFL	Increased thickness in the acute phase of optic neuritis	Costello et al.^39^
	Chronic thinning in patients with and without optic neuritis	Parisi et al.^40^, Petzold et al.^41^, Garcia-Martin et al.^42^
	Thinning is correlated with visual loss	Trip et al.^43^
Microvascular	Reduction in the retinal and macular vessel densities	Lanzillo et al.^45^, Lanzillo et al.^46^, Spain et al.^47^, Wang et al.^48^
Parkinson’s disease		
pRNFL	Inconsistent findings	Inzelberg et al.^52^, Altintas et al.^53^, Hajee et al.^54^, Albrecht et al.^55^, Mailankody et al.^56^, Lee et al.^57^
	Thinner RNFL in akinetic-rigid Parkinson’s disease, compared with tremor-predominant type	Altintas et al.^53^
	Negative correlation between UPDRS and RNFL	Albrecht et al.^55^, Mailankody et al.^56^, Moschos et al.^59^
	Correlation between pRNFL thinning and nonmotor symptoms	Lee et al.^57^, Yildiz et al.^60^, Ma et al.^61^, Satue et al.^62^
Macula	Decreased volume and thickness	Altintas et al.^53^, Hajee et al.^54^
	Ganglion cell-inner plexiform layer abnormalities	Chrysou et al.^63^
Amyotrophic lateral sclerosis		
pRNFL	Diffusely reduced thickness, compared with controls	Rohani et al.^72^, Rojas et al.^73^
Macula	Thinning in outer and inner sectors	Rojas et al.^73^
Microvascular	Inconclusive findings	Cervero et al.^75^

OCT: optical coherence tomography; pRNFL: peripapillary retinal nerve fiber layer; MoCA: Montreal Cognitive Assessment; MMSE: Mini-Mental State Examination; RNFL: retinal nerve fiber layer; UPDRS: Unified Parkinson’s disease rating scale.

However, we need to address some drawbacks regarding this technology. First, the use of OCT to detect neuroaxonal loss, seen in patients with neurodegenerative diseases, may be influenced by the presence of other ocular diseases that are prevalent in the elderly population, such as glaucoma and age-related macular degeneration. Moreover, other previous ocular diseases can also provide impaired results that do not have any correlation with the neurodegenerative disease itself.

Second, normative data from OCT devices has the clinical purpose of helping doctors to recognize an abnormal OCT examination and this involves volumetric analyses on the pRNFL and macular thickness values. In some neurodegenerative conditions, especially in the very early phases, OCT may exhibit values within normal limits. To the best of our knowledge, the individuals who were included for the normative databases were not screened to exclude those who had family histories of neurodegenerative disorders or who had previously undergone cognitive tests. This might have impacted on the diagnostic ability to detect neuronal loss, especially in the very early phases and even in borderline cases. We believe that it is necessary for physicians to take into account analyses on OCT data values ​​from patients with neurodegenerative diseases, based on published studies on these specific populations. We also recommend that companies supplying OCT technology should maintain awareness of these limitations and look for solutions to mitigate this problem, thus improving the diagnostic capability of OCT in relation to these specific groups of diseases.

Another important suggestion is that, after a certain age, especially in cases with a higher risk of onset of a neurodegenerative disease, patients should undergo serial analysis using OCT, to observe whether any significant reduction of these parameters might occur ​​over the years.

Another major concern, especially in developing countries such as Brazil, is the limited number of OCT tests that are done in socioeconomically vulnerable populations. OCT tests need to be more available for population screening.

In conclusion, OCT is a handy noninvasive tool for diagnosing and following neuroaxonal loss in many neurodegenerative diseases and could be potentially used to provide an easily accessible ocular biomarker in these patients.
